# Improvement of the YOLOv8 Model in the Optimization of the Weed Recognition Algorithm in Cotton Field

**DOI:** 10.3390/plants13131843

**Published:** 2024-07-04

**Authors:** Lu Zheng, Junchao Yi, Pengcheng He, Jun Tie, Yibo Zhang, Weibo Wu, Lyujia Long

**Affiliations:** 1 College of Computer Science, South-Central Minzu University, Wuhan 430074, China; 2 Hubei Provincial Engineering Research Center of Agricultural Blockchain and Intelligent Management, Wuhan 430074, China; 3 Hubei Academy of Scientific and Technical Information, Wuhan 430071, China

**Keywords:** cotton weed, YOLOv8, Multi-Scale module, ASFF, intelligent weeding

## Abstract

Due to the existence of cotton weeds in a complex cotton field environment with many different species, dense distribution, partial occlusion, and small target phenomena, the use of the YOLO algorithm is prone to problems such as low detection accuracy, serious misdetection, etc. In this study, we propose a YOLOv8-DMAS model for the detection of cotton weeds in complex environments based on the YOLOv8 detection algorithm. To enhance the ability of the model to capture multi-scale features of different weeds, all the BottleNeck are replaced by the Dilation-wise Residual Module (DWR) in the C2f network, and the Multi-Scale module (MSBlock) is added in the last layer of the backbone. Additionally, a small-target detection layer is added to the head structure to avoid the omission of small-target weed detection, and the Adaptively Spatial Feature Fusion mechanism (ASFF) is used to improve the detection head to solve the spatial inconsistency problem of feature fusion. Finally, the original Non-maximum suppression (NMS) method is replaced with SoftNMS to improve the accuracy under dense weed detection. In comparison to YOLO v8s, the experimental results show that the improved YOLOv8-DMAS improves accuracy, recall, mAP0.5, and mAP0.5:0.95 by 1.7%, 3.8%, 2.1%, and 3.7%, respectively. Furthermore, compared to the mature target detection algorithms YOLOv5s, YOLOv7, and SSD, it improves 4.8%, 4.5%, and 5.9% on mAP0.5:0.95, respectively. The results show that the improved model could accurately detect cotton weeds in complex field environments in real time and provide technical support for intelligent weeding research.

## 1. Introduction

Cotton, known as ‘White Gold’ or ‘The King of Fibers’ [1], was a significant cash crop that provided a lucrative income to millions of farmers in both developed and developing countries [2]. However, cotton faced significant threats from pests, disease infestations, and weed competition. Weed competition was a biotic stress problem that severely limited crop growth by competing for resources such as water and sunlight. Additionally, weeds could act as a medium for the spread of pests and diseases, causing significant damage to crop yields. Globally, weed stress was estimated to cause 43% of crop yield losses [3]. The development of precise and effective technology for detecting cotton weeds would be essential for safeguarding cotton yield.

Weed control was a persistent issue in agriculture [4]. Farmers employed various strategies to remove weeds, including mechanical methods such as mowing or plowing and chemical methods such as herbicides [5]. Although herbicides were the most commonly used method, over-reliance on them for weed management could lead to the rapid evolution of herbicide-resistant weeds. Consequently, the intelligent use of advanced computer vision techniques for weeding has attracted extensive research.

Originally, machine learning classifiers were used for weed identification to distinguish them from crops. In their study, Zhao et al. [6] used Hu invariant moments to obtain soybean leaf features. They then recognized these features using a nearest neighbor classifier with 90.5% accuracy in a normal environment. Lavania et al. [7] proposed a weed detection model that combined the 3D-Otsu and PCA methods. This model could detect weeds in crop rows with 91.67% accuracy. Bakhshipour et al. [8] used support vector machines and artificial neural networks to study four common weeds in sugar beet fields. The overall classification accuracy of the artificial neural network was 92.92%. Zhang et al. [9] presented a method for identifying weed species that combines improved Grabcut, an adaptive fuzzy dynamic K-means algorithm, and Sparse Representation Classification (SRC). The method achieved 95% accuracy on a self-made dataset in a wheat field.

Although machine learning could produce promising results in weed classification, it heavily relied on manual feature extraction. This approach overlooked some critical image features and failed to capture hidden patterns in the data. In contrast, deep learning can often achieve better classification outcomes than machine learning algorithms [10]. In their study, Hung et al. [11] employed CNN to accurately classify three types of weeds, achieving accuracies of 72.2%, 92.9%, and 94.3% for each weed. Chavan et al. [12] designed the AgroAVNET model by fusing AlexNet and VGGNet using the Plantseedling dataset. The model achieved a classification accuracy of 93.64% in single-object recognition, where only one weed would be presented in each image. Abdalla et al. [13] studied the effectiveness of detecting semantic segmentation of weeds in high-density vegetation and achieved an accuracy of 96% by using a pre-trained VGG16 network. Ferreira et al. [14] investigated clustered CNN algorithms with labeled and unlabeled data to reduce the labeling workload. The algorithm achieved good performance in semi-automatic weed identification on labeled data. Zhao et al. [15] proposed an improved DenseNet weed identification model for identifying companion weeds for maize seedlings in a natural environment. The model achieved an average identification accuracy of 98.63% by introducing Efficient Channel Attention (ECA) and DropBlock regularization. Yang et al. [16] introduced a lightweight weed recognition method by improving the MobileViT network. The method achieved 99.61% accuracy in four types of companion corn weed datasets, with a single recognition taking only 83 ms.

Mainstream object detection algorithms could be classified as one-stage or two-stage based on their processing flow. In the two-stage algorithm exemplified by R-CNN, the object’s location was first determined to ensure accuracy, and then feature extraction and classification were performed to precisely locate the object in the second stage. As a classical one-stage algorithm, YOLO used a CNN to directly obtain the input image’s target class and location predictions [17]. Only a single detection was needed to obtain the final result. Peng et al. [18] introduced the WeedDet model based on RetinaNet, which attained a high mean Average Precision (mAP) of 94.1% and a frame rate of 24.3 fps on a self-built paddy field weed dataset. In Sharpe et al.’s study [19] on goose grass detection, the improved YOLOv3-tiny model achieved an F-1 score of 0.85 and 0.65 in strawberry and tomato fields, respectively. Additionally, Gao et al. [20] proposed a simplified version of YOLOv3-tiny for detecting hedge weeds in a beet field, achieving a mean Average Precision (mAP) of 0.829. Ahmad et al. [21] utilized YOLOv3 to identify four prevalent weed species in the Midwest of the United States, achieving an overall mAP of 54.3%. Zhao et al. [22] proposed the MC-YOLOv4 model, which replaced CSPDarkNet53 in the YOLOv4 network with the lightweight MobileNet v3 network and applied an attention mechanism to enhance feature extraction ability for identifying weeds in potato fields. This resulted in a 3.2% improvement in the mAP value of YOLOv4. Shao et al. [23] developed an enhanced deep learning model named GTCBS-YOLOv5s to identify six types of weeds in rice paddies, demonstrating satisfactory performance under various lighting conditions. Furthermore, García-Navarrete et al. [24] implemented a corn weed detection system based on the YOLOv5 model, achieving an mAP@0.5 (mean Average Precision) of 83.6% at a threshold of 0.5 for the trained model. Zhu et al. [25] proposed an improved YOLOX model for corn weed detection, achieving an average detection accuracy of 94.86% by integrating lightweight attention mechanisms with deep network connections. In cotton weed detection, Peng et al. [26] used a Faster R-CNN integrated with FPN, achieving an average target recognition accuracy of 95.5% and an average processing time of 1.51 s per image.

Currently, weed detection algorithms based on convolutional neural networks face several challenges. One of the main issues was the inability of most models to strike a balance between detection speed and accuracy, leading to numerous false detections and missed targets. Additionally, most models could only identify a limited number of weed categories, and the size of weeds was often similar, further complicating the detection process. We proposed an improved YOLOv8-DMAS algorithm to address the challenges of accurately detecting small and partially covered weeds, which was crucial for intelligent weeding in complex situations. This provided a technical reference for automated weed removal in cotton fields. The main contributions of this paper would be as follows:

(1) Given the challenge posed by the diverse types of cotton weeds and the significant variation in the sizes of different weed species, the C2f network was enhanced through the integration of the Dilation-wise Residual Module (DWR) in place of the conventional BottleNeck structure. Additionally, a Multi-Scale module (MSBlock) was incorporated following the Spatial Pyramid Pooling—Fast (SPPF). These modifications were designed to augment the model’s capability in extracting multi-scale features and to bolster the model’s generalization capacity for multi-scale contextual information, achieved by broadening the receptive field.

(2) To enhance the detection of smaller weed targets, a specialized small-target detection head was added to the original YOLOv8 framework. Furthermore, to mitigate the issue of multi-scale feature conflicts across different detection heads, an adaptive spatial feature fusion mechanism (ASFF) was implemented throughout all detection heads. This approach aimed to harmonize the integration of multi-scale features, ensuring a more cohesive and conflict-free feature representation.

(3) To address the challenges associated with detecting dense and partially occluded weed samples, the conventional NMS method was substituted with SoftNMS. This modification was intended to significantly improve the detection performance of the model, offering enhanced precision in identifying and differentiating between closely situated or overlapping weed instances.

## 2. Results and Discussion

### 2.1. Experimental Platform

The experiments were performed on the cloud server. To ensure the unity of the experimental environment, the model training and testing experiments used the same platform. The hardware configuration was a 16-core AMD EPYC 7532 @2.4 GHz CPU and an NVIDIA GeForce RTX A6000 48 GB. The operating system was Ubuntu 18.04, the deep learning framework was pytorch 1.11.0, the programming platform was Pycharm, Compute Unified Device Architecture (CUDA) 12.1, and python 3.8 was used for programming.

The training configuration parameters of the network were as follows: the image input size was 640 × 640, the initial momentum of Stochastic Gradient Descent (SGD) was set to 0.937, the batch size was set to 16, the weight decay coefficient was set to 0.0005, the learning rate lr = 0.01, and the cosine decay rate scheduler was used. After 300 epochs of training, the optimal weight model was obtained. The Warmup Strategy Preheating was performed in the first three epochs.

### 2.2. Performance Evaluation of Models

In this study, we used precision (P), recall (R), F1-score, mean Average Precision (mAP), FPS, FLOPs, and parameter sizes to evaluate the model’s performance. Precision was the probability of correct detection among all detected targets. And Recall was the probability of correct identification among all positive samples. The F1-score measured the combined performance of precision and recall. Usually used as the final metric for model evaluation. By computing the accuracy and recall of the target detection network on the test set under different confidence thresholds, we can obtain the Precision-Recall (P-R) curve. Utilizing the Average Precision (AP) evaluates the performance of the defect detection model, where this precision (AP) denotes the area under the P-R curve. While mAP averaged the AP from the dimensions of the categories, it could thus evaluate the performance of multiple classifiers. These metrics would take the value in the interval [0, 1], and the higher the value, the better the detection result represented. The above five indices are mathematically expressed in Equations (1) to (5):

(1) $$\mathrm{Precision}=\frac{\mathrm{TP}}{\mathrm{TP}+\mathrm{FP}}$$

(2) $$Recall=\frac{TP}{TP+FN}$$

(3) $$F1\text{-}score=\frac{2\times Precision\times Recall}{Precision+Recall}$$

(4) $$AP=\int_{0}^{1}\;p(r)dr$$

(5) $$mAP=\frac{AP}{classes\_num}$$

where TP meant that both true and predicted values were positive, and TN meant that both were negative. FP meant that the true value would be negative and the predicted value was positive; FN meant that the true value would be positive and the predicted value was negative. The classes_num was the number of detected classes.

### 2.3. Ablation Experiments

To verify the performance of the improved YOLOv8 model, we set up an ablation experiment to verify the performance of the five sets of networks. The YOLOv8s model was used as a baseline to sequentially add the DWR module, the Multi-Scale module, the improved detection header, and the SoftNMS algorithm. We analyzed the performance of five groups of networks from a quantitative perspective and performed a comprehensive evaluation of 848 cotton weed images, including 12 categories in the test set. The evaluation metrics contained P, R, mAP0.5, mAP0.5:0.95, model parameter size, and GFLOPS. The experimental results are shown in Table 1.

Table 1 showed that the change in each module improved the mAP of the original YOLOv8s model. The use of the DWR module to replace Bottleneck reduced the number of parameters and GFlops by 5.4% and 4.2%, respectively. However, there was a small increase in all metrics except recall. This demonstrated that the DWR module could enhance the multi-scale feature extraction capability of the model but remained deficient. The model metrics were all greatly improved by including MSBlock, which allowed for efficient multi-scale feature extraction. However, the number of parameters and GFlops increased. The difficulty in improving model recognition accuracy and small target detection is considered because of the conflicting information between model multi-scale information. We added the ASFF mechanism and small target detection head, and the P, R, mAP.5, and mAP0.5:0.95 were improved by 1.2%, 2.7%, 1.1%, and 3%, respectively. This indicated that the method fully utilized the advantages of multi-scale extraction of the model. Eventually, the SoftNMS method was replaced to address the difficult samples for dense detection. The final improved model outperformed the original model by 2.1% and 3.7% in mAP0.5 and mAP0.5:0.95, which was a very significant advantage in weed detection tasks.

### 2.4. Comparison Experiments

In this study, a series of improvements were applied to the original YOLOv8s model with the aim of improving the accuracy and efficiency of weed target detection. To evaluate the performance of the improved model, we selected nine different detection algorithms for comparison, and used the test set without data augmentation for verification. The experiment followed the principle of control variables to ensure a consistent experimental environment. After 300 epochs, all models converged stably.

Table 2 shows the experimental results of YOLOv3, YOLOv4-tiny, YOLOv5s, YOLOv7, YOLOv8s, YOLOv8l, SSD [27], Faster R-CNN [28], and YOLOv8-DMAS. The result showed that the YOLOv8 model significantly outperforms the other single-stage algorithms and the two-stage algorithms with higher mAP and less single-frame detection time. Although there was a small increase in the number of parameters in our improved network structure based on YOLOv8s compared to the original model, it had the highest mAP, accuracy, and recall among all models. Furthermore, the new model outperformed the YOLOv8l model in all three metrics: number of parameters, GFLOPS, and FPS. YOLOv8-DMAS obtained better recognition results than YOLOv8l using only about 43.61% of the parameters, outperforming all mainstream models. The improvement of cotton weed detection in terms of recognition accuracy and speed was proven to be effective.

**Figure 1:**
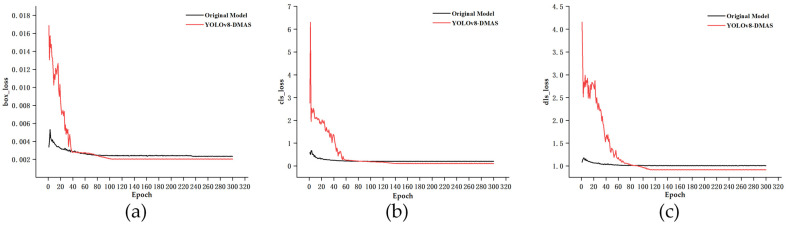
Loss function curve: (**a**) comparison of the original and improved model’s box Loss over Epochs, (**b**) comparison of the original and improved model’s Object Loss over Epochs, and (**c**) comparison of the original and improved model’s class Loss over Epochs. The term “epoch” refers to the completion of one full cycle of sending all data into the network, performing forward calculation and backpropagation processes, with its unit measured in times.

The loss variation curves of YOLOv8s and the optimized YOLOv8 model during training are shown in Figure 1. We could see that the loss of the original model was much smaller than the improved model at the beginning due to the use of pre-training weights, but both have started to converge after 100 epochs. In contrast, the YOLOv8-DMAS model had a violent oscillation of loss in the initial stage and gradually converged after 120 rounds. As seen from the overall loss function curves, there were no phenomena such as gradient explosion or disappearance during the training process. In addition, the loss of the improved model was lower than that of the original model, which further indicated that the improved model had more powerful detection abilities.

### 2.5. Visual Analysis of Test Results

Visualization provided a more intuitive view of the effectiveness of the algorithm’s detection, allowing the observation of information about the location, size, and class of targets detected by the algorithm. This could be used to evaluate the accuracy and efficiency of the algorithm, as well as to debug and optimize it. In addition, the detection results can be used for data analysis and research, like analyzing the distribution and morphological characteristics of different classes of targets in an image. This could lead to a better understanding of the performance and application scenarios of the target detection algorithms and further improve the accuracy of the algorithms.

We validated cotton weed detection in different scenarios, including dense weeds, partial occlusion, multiple species detection, small-volume weeds, and other complex situations. The detection results were also compared.

**Figure 2:**
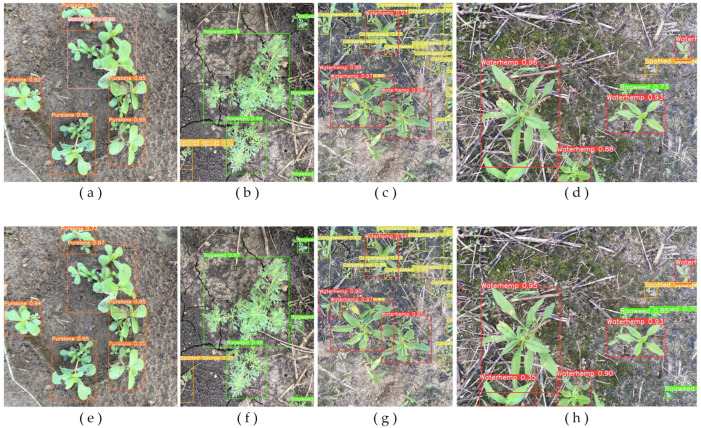
Detection results for selected images. For each column of images, a top-to-bottom comparison was conducted. The first row (**a**–**d**) shows the detection results of YOLOv8s, and the second row (**e**–**h**) shows the detection results of YOLOv8-DMAS.

Figure 2 illustrates some detection results, with the first row showing the original YOLOv8s and the second row showing the improved model. In Figure 2a, due to the large number of the same weeds, which largely differed in size, the original model incorrectly classified the largest weeds. The improved model, however, could detect them accurately and efficiently, as shown in Figure 2e. In Figure 2b, the weeds in the lower left corner were detected repeatedly by the original model because they only partially existed in the image. This increased the possibility of false detection. Figure 2c showed the difficult samples in cotton weeds; we could see that the original model suffered many iterative detections of a single target in dense detection scenarios, and our model avoided this problem. In Figure 2d, the weeds in the lower right corner were ignored by the small size, and the partially occluded weeds in the lower left corner were also not detected. The results showed that the original model had difficulty detecting small weeds, dense and partially occluded weeds, and multi-scale weeds. Thus, the feature extraction capability of the model was defective.

The YOLOv8-DMAS model proposed in this paper solved these problems, improved the confidence level of detection, and demonstrated the effectiveness of the model for weed detection tasks in complex cotton fields.

To reveal the black box effect of deep learning models, we used the Grad-CAM [29] heat map visualization method proposed by Inbaraj et al. to draw heat maps of the images detected by YOLOv8s and YOLOv8-DMAS in the end. Grad-CAM was the classic method for interpreting CNN. The method calculated the importance of the feature map of a convolutional neural network on the output categories and visualized the result into a heat map, and this was used to interpret the network’s prediction results for the input image. This principle helped to explain the inner patterns of convolutional neural networks, thereby improving the interpretability and reliability of the network.

**Figure 3:**
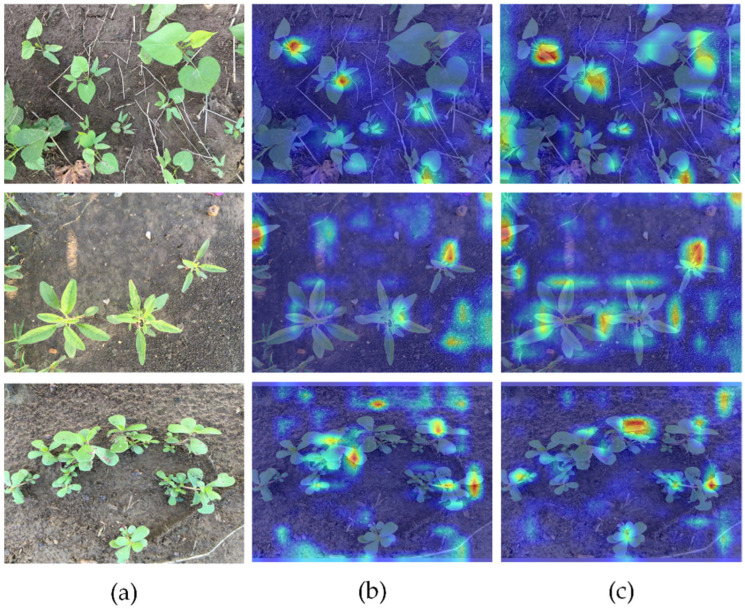
Heat map of test results: (**a**) input images, (**b**) YOLOv8s, and (**c**) YOLOv8-DMAS.

As shown in Figure 3, YOLOv8s tended to focus more on medium, large, and distinctive weeds, but it was unable to spread its attention to all the weeds that needed detection. Small weeds and weeds at the edge of the image were also easily ignored. In contrast, the YOLOv8-DMAS could focus more precisely on all areas. This meant it could better learn the multi-scale features and information in the image and improve detection accuracy. The method was highly significant for cotton weed detection in practical scenarios.

## 3. Materials and Methods

### 3.1. Cotton Weed Dataset

#### 3.1.1. Data Selection

**Figure 4:**
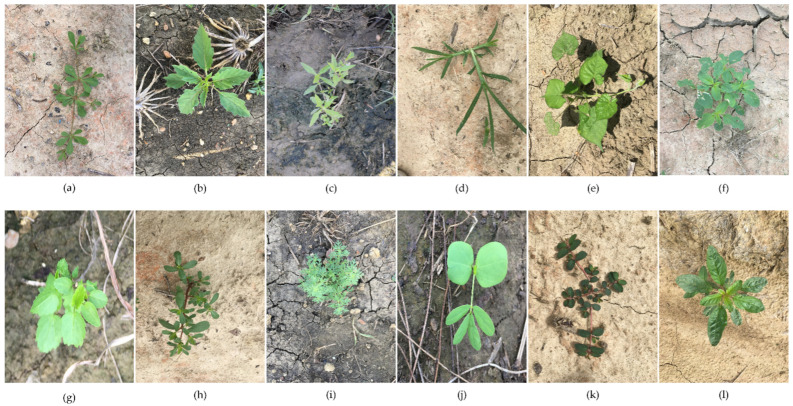
Cotton field 12 types of weeds: (**a**) Carpetweed, (**b**) Cutleaf GroundCherry, (**c**) Eclipta, (**d**) Goosegrass, (**e**) Morningglory, (**f**) Palmer Amaranth, (**g**) Prickly Sida, (**h**) Purslane, (**i**) Ragweed, (**j**) Sicklepod, (**k**) Spotted Spurge, and (**l**) Waterhemp.

The public cotton weed dataset CottonWeedDet12 [30] utilized in this research was collected from cotton fields of Mississippi State University research farms, including the R. R. Foil Plant Science Research Center (Starkville, MS, USA), W.B. Andrews Agricultural Systems Research Farm (Starkville, MS, USA), and Black Belt Experiment Station (Brooksville, MS, USA). During the data collection phase, images within the dataset were captured by mobile devices equipped with cameras of varying high-resolution pixels. This approach was deliberately chosen to mirror the complex scenarios encountered in cotton fields during the model’s deployment phase, encompassing a variety of weed growth stages, lighting conditions, weather conditions, and field locations. Such a comprehensive collection strategy ensured the dataset’s diversity and complexity, thereby mitigating the risk of model overfitting throughout the training process. In the annotation phase, the VGG image annotator was employed to delineate bounding boxes, generating both JSON and txt formats for data storage. Through meticulous data filtering, a robust cotton weed dataset was compiled, featuring 12 distinct categories of cotton weeds and encompassing 5648 images and 9370 bounding box annotations. The categories of weeds are depicted in Figure 4. Their Latin names were *Trigastrotheca stricta (Carpetweed)*, *Physalis angulate (Cutleaf GroundCherry)*, *Eclipta prostrata (Eclipta)*, *Eleusine indica (Goosegrass)*, *Ipomoea nil (Morningglory)*, *Amaranthus palmeri (Palmer Amaranth)*, *Eryngium foetidum (Prickly Sida)*, *Portulaca oleracea (Purslane)*, *Ambrosia artemisiifolia (Ragweed)*, *stag beetle (Sicklepod)*, *Euphorbia maculate (Spotted Spurge)*, and *Debregeasia orientalis (Waterhemp)*. The English names of the Latin names are presented in parentheses.

#### 3.1.2. Image Augmentation

**Figure 5:**
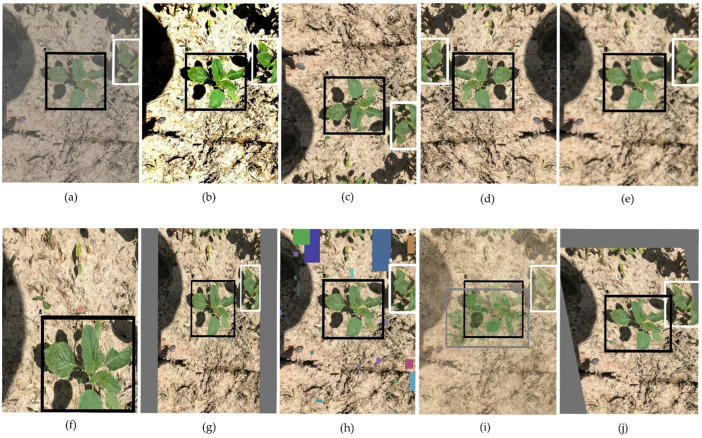
Weed image enhancement. (**a**) HLs-Dark, (**b**) HLs-Light, (**c**) Flip Vertical, (**d**) Turn Around, (**e**) Mean Blur, (**f**) Random Crop, (**g**) Random Resize, (**h**) Random Noise, (**i**) Distortion, and (**j**) Mix Up.

The CottonWeedDet12 dataset was rich in weed species, but the total number of samples was still lacking. This could make it difficult for the model to converge or even result in overfitting during the training process. Additionally, there were many difficult samples in the dataset with small targets and mutual occlusions. The model also struggled to perform the generalization ability to predict that portion of the difficult samples in the case of insufficient data. To solve the above problems, we first divided the data set into a training set, a validation set, and a test set in the ratio of 70%:15%:15% on the dataset. The training set, test set, and validation set were 3953, 848, and 847 images, respectively. Next, for the training set, we randomly expanded each image by using ten different enhancement methods, as shown in Figure 5. The specific methods included: HLs-Dark (reducing brightness and contrast), HLs-Light (increasing brightness and contrast), Flip Vertical (vertical flip), Turn Around (rotating 90, 180, or 270 degrees), Mean Blur (mean blur), Random Crop (random cropping), Random Resize (random resizing), Random Noise (adding random noise), Distortion (random distortion), and Mix Up (image mixing). The different colored bounding boxes in the images represented different categories, and in some data augmentation methods (Random Crop, Random Resize, and Mixup), the bounding boxes also changed. Finally, the amount of the training set was expanded to twice the original number, leading to a final dataset of 9602 images. This increased the complexity of the data and enabled the model to be trained more effectively with improved generalization and robustness. Table 3 showed detailed information about the augmented dataset category, image size, division ratio, and number of samples.

### 3.2. YOLOv8 Model

**Figure 6:**
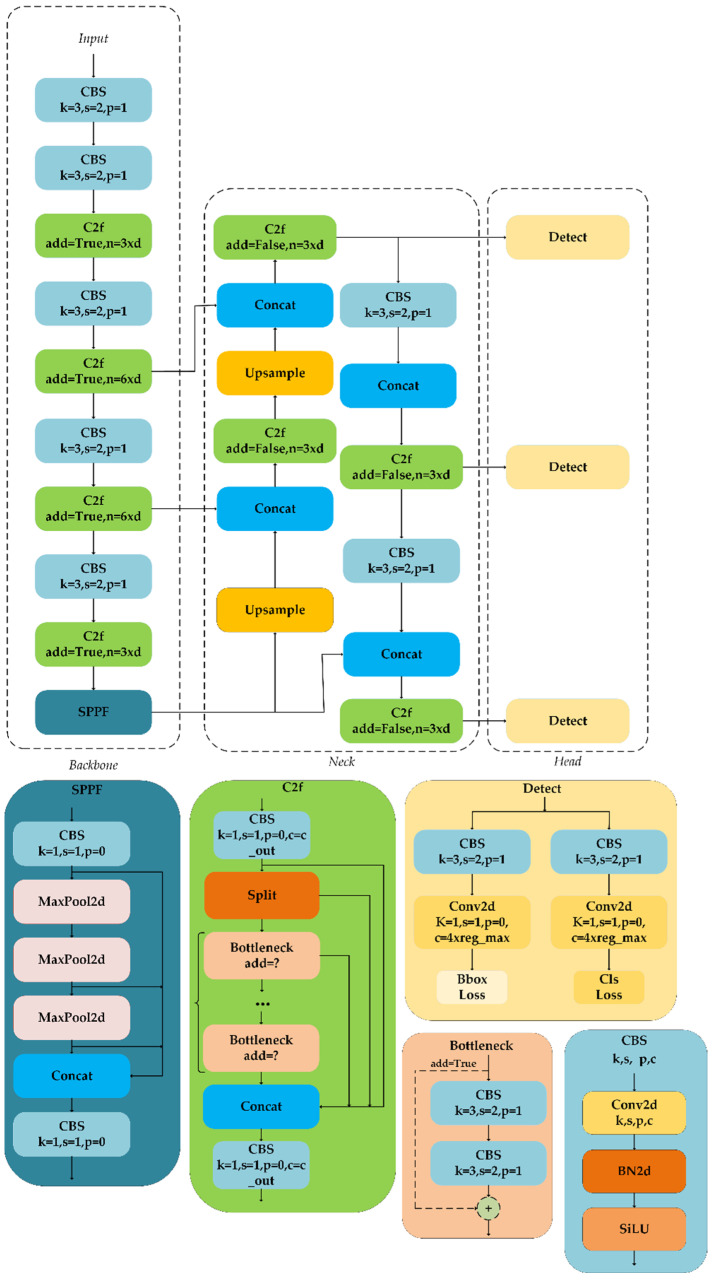
YOLOv8 model structure.

In this study, YOLOv8 was selected as the latest version of the YOLO object detection and image segmentation model developed by Ultralytics, based on YOLOv5. This model, trained from scratch on the COCO dataset, surpassed detectors such as YOLOv7 [31], YOLOR [32], YOLOX [33], and Scaled-YOLOv4 [34] in terms of detection speed and accuracy, making it the state-of-the-art (SOTA) model for fast and efficient detection within the YOLO series. YOLOv8 included five different versions of increasing parameter sizes. We optimized YOLOv8s due to its characteristics of having fewer parameters, high accuracy, and fast detection speed, making it suitable for cotton weed detection. The use of an excessive number of transition layers leads to a continual increase in the shortest gradient path [35]. In terms of model architecture, YOLOv8, drawing on v7, introduced a new efficient network architecture, C2f, replacing the C3 network of YOLOv5. By controlling the longest and shortest gradient paths, this architecture improved the computational block’s network structure, enabling the model to learn more features and exhibit stronger robustness. Additionally, the head design was modified by replacing the coupled head with a decoupled head structure to separate classification and detection, thereby enhancing accuracy. Furthermore, improvements were made over YOLOv5 in aspects, such as the positive and negative sample matching strategy, loss function, and data augmentation, as shown in Figure 6.

### 3.3. Improved YOLOv8-DMAS

**Figure 7:**
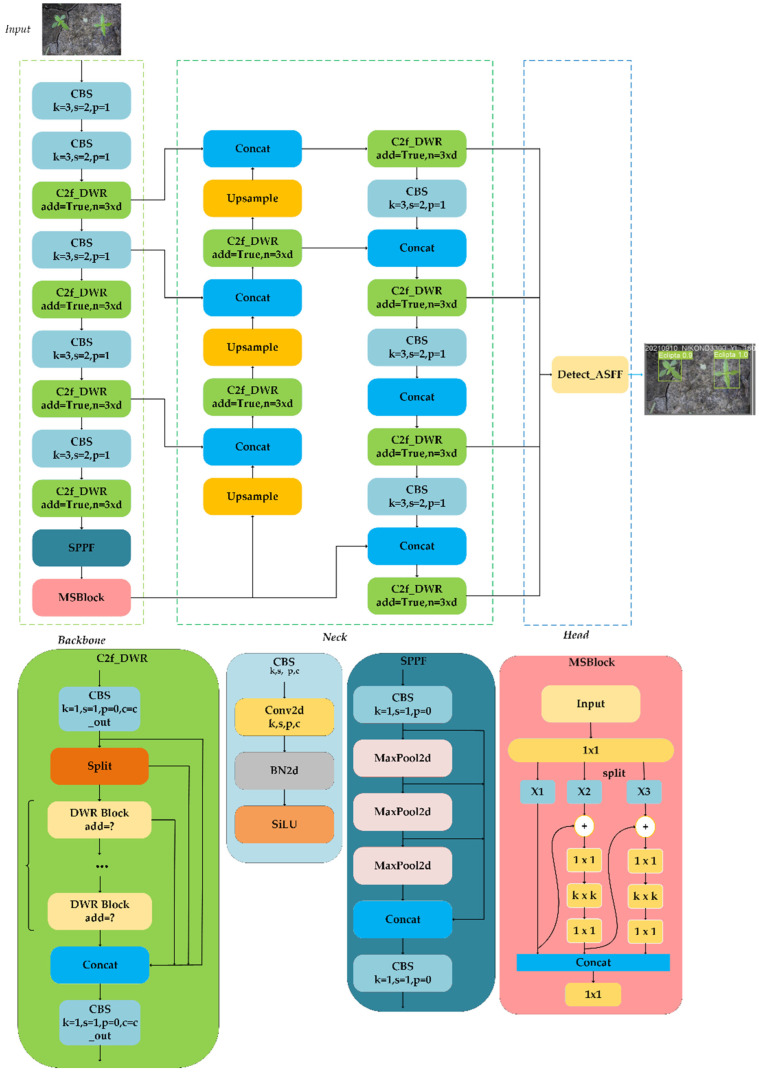
Improved YOLOv8 model structure.

Although the original YOLOv8 model significantly enhanced detection accuracy, it underperformed in densely populated detection scenarios, leading to both misdetections and missed detections. Additionally, its generalization capability across various datasets fell short of fulfilling practical requirements. Within the CottonWeedDet12 dataset, which hosted a diverse range of cotton weed species across different scales, the model’s performance became compromised by interference from large volume weeds, resulting in overlooked small target weeds. Therefore, we proposed to improve the original model in four ways. The improved network structure is shown in Figure 7. First, the DWR module replaced the BottleNeck structure of the C2f network, and the Multi-Scale Block, a hierarchical feature fusion module, was added at the end of the backbone to obtain the multi-scale feature information of cotton weeds. Next, we added a small-target detection head to improve the detection of small-target weeds. Then, we added an adaptive spatial feature fusion mechanism (ASFF) in the detection head to filter contradictory features. Eventually, the conventional non-maximum suppression (NMS) method was replaced with SoftNMS, which improved the performance of dense weed detection. According to the different modules improved by the model, it was named YOLOv8-DMAS. The network architecture of YOLOv8-DMAS is illustrated in Figure 7. Specific improvements would be described below.

#### 3.3.1. DWR Module

**Figure 8:**
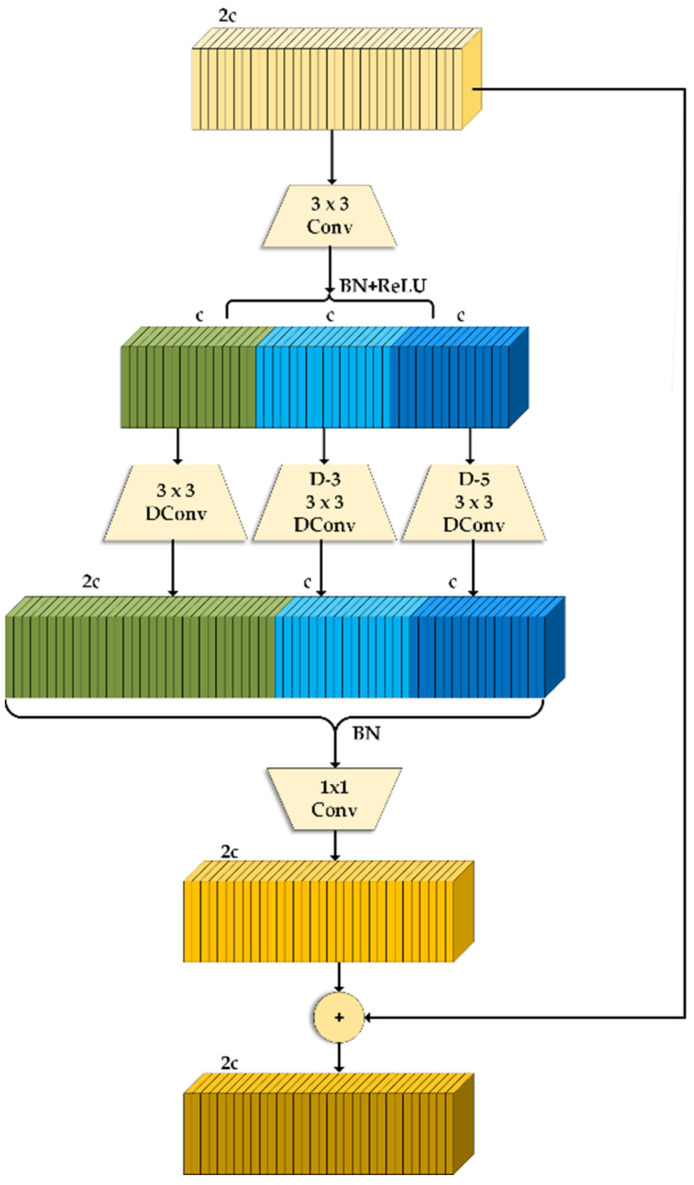
DWR module structure diagram.

The DWR module proposed by DWRSeg [36] was a multi-branch dilation-wise residual convolution block. The module was designed in a residual manner, as shown in Figure 8, where multi-scale context information was efficiently drawn in some stages within the residual, and the feature maps generated by multi-scale sensory fields were fused. In the first stage, a 3 × 3 size standard convolution was used for preliminary extraction to generate a series of concise feature maps of regions of different sizes. In the second stage, three branches using deep dilated convolution with hole rates of 1, 3, and 5 were set to analyze the semantic information of the feature maps of different scales in the first stage. Then, all branches were connected. Regardless of the network stage, features extracted with smaller receptive fields were relatively important because feature connections with large spatial distances always required smaller intermediate features to be established. Therefore, the number of channels in the dilated convolution branch with the lowest dilation rate was set to twice that of the other branches. Eventually, feature fusion was performed using pointwise convolution, and the output was added to the input feature map to build a more comprehensive feature representation.

The receptive fields varied at different stages of the network, and the requirements for different sizes of receptive fields within the same layer were also distinct. This module was aimed at solving the problem of incomplete feature extraction due to the large number of cotton weed species and the great difference in their volume. Consequently, efficient multi-scale feature extraction could be performed for different weeds.

#### 3.3.2. Multi-Scale Block

**Figure 9:**
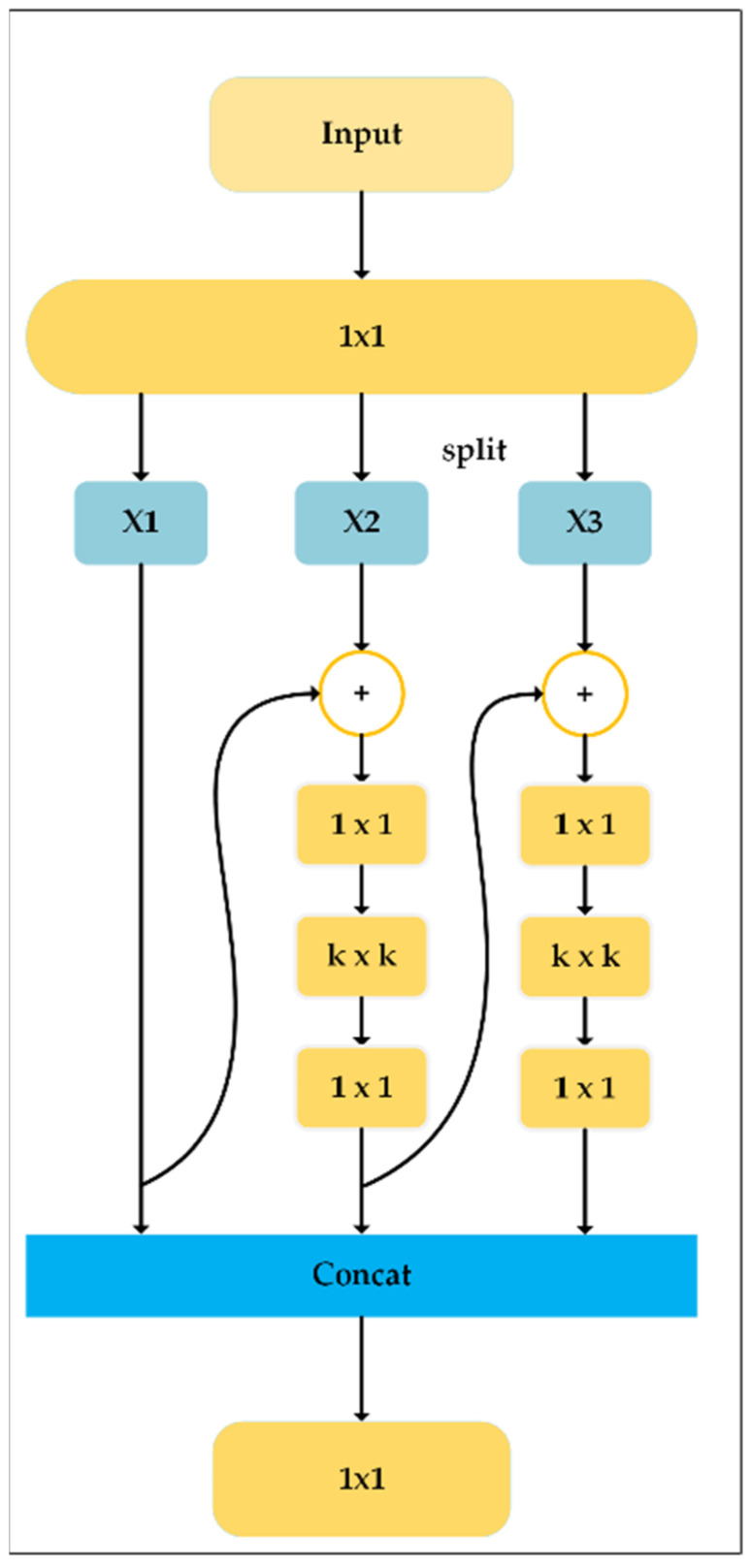
Multi-Scale Block structure diagram.

To make up for the lack of feature extraction in the DWR module and further enhance the multi-scale feature representation of the model, we introduced the Multi-Scale Block. Chen et al. proposed a hierarchical feature fusion module in YOLO-MS [37]. Its structure is shown in Figure 9. This module performed feature extraction by introducing a multi-branch structure and effectively using large convolution kernels. A wider receptive field was provided to construct multi-scale features.

Suppose the input features is $\begin{array}{c} X\in R^{H\times W\times C} \end{array}$. First, the number of its channels was increased by 3 times the original input using 1 × 1 convolution, and then X was divided into 3 groups to reduce the computational cost, denoted as $\left\{X_{i}\right\},i\in 1,2,3.$ Except for $X_{1}$, the other two groups had to pass through a reverse bottleneck layer (denoted as $IB_{9\times 9}(\cdot )$) to obtain $F_{i}$, and different groups encode different scale features. The mathematical expression for $F_{i}$ was as follows in Equation (6):

(6) $$\left.F_{i}=\left\{\begin{array}{ll} X_{i}, & i=1\\ IB_{9\times 9}(F_{i-1}+X_{i}). & i>1 \end{array}\right.\right.$$

Finally, all groups were connected according to channels, and a 1 × 1 convolution was used to interact between groups. To extract fine-grained and coarse-grained semantic information at the same time, we added it after spatial pyramid pooling to enhance the multi-scale feature representation ability of the model. Since large kernel convolution for low-resolution feature maps could reduce the computational cost, 9 × 9 convolution was chosen here as the intermediate layer of this module.

#### 3.3.3. Improved Detection Head

YOLOv8 employed three detection heads to process feature maps at three distinct scales: 20 × 20 pixels, 40 × 40 pixels, and 80 × 80 pixels. However, within the cotton weed dataset, certain weed species were small, posing challenges for the original model, which might fail to detect or accurately identify such targets. To address this issue, we introduced an additional detection head specifically designed for small objects. This new detection head received input from the feature maps produced by the first C2f module of the backbone network, combined with 160 × 160-pixel feature maps obtained after three stages of upsampling. Since this detection layer integrated the shallow information from the backbone network, it was enriched with abundant low-level details, significantly enhancing the model’s capability to generalize and accurately detect small-scale weed species. Additionally, since we used multiple multi-scale feature extraction strategies, the inconsistency of the learning objectives among different scale features was more likely to arise during the allocation process of the feature pyramid network. In this regard, we introduced the adaptive spatial feature fusion mechanism (ASFF) [38] to improve the detection head. For the input from a single detector head, the inputs from the other heads were resized by upsampling or downsampling to the size of the feature map for this head, and then weighted sum. This was expressed in Equation (7) as follows:

(7) $$y_{ij}^{l}=\alpha_{ij}^{l}\cdot x_{ij}^{1\to l}+\beta_{ij}^{l}\cdot x_{ij}^{2\to l}+\gamma_{ij}^{l}\cdot x_{ij}^{3\to l}+\lambda_{ij}^{l}\cdot x_{ij}^{4\to l}$$

where $\alpha_{ij}^{l}+\beta_{ij}^{l}+\gamma_{ij}^{l}+\lambda_{ij}^{l}=1$ and $\alpha_{ij}^{l},\beta_{ij}^{l},\gamma_{ij}^{l},\lambda_{ij}^{l}\in [0,1]$, $y_{ij}^{l}$ denotes the output features of layer l, and $x_{ij}^{s\to l}$ denotes the feature map from layer s resize to layer t. α, β, γ are the learnable parameters, indicating the importance of different levels of feature maps. $\alpha_{ij}^{l}$, $\beta_{ij}^{l}$, $\gamma_{ij}^{l}$ denote the values of α, β, γ at position (i, j), respectively.

**Figure 10:**
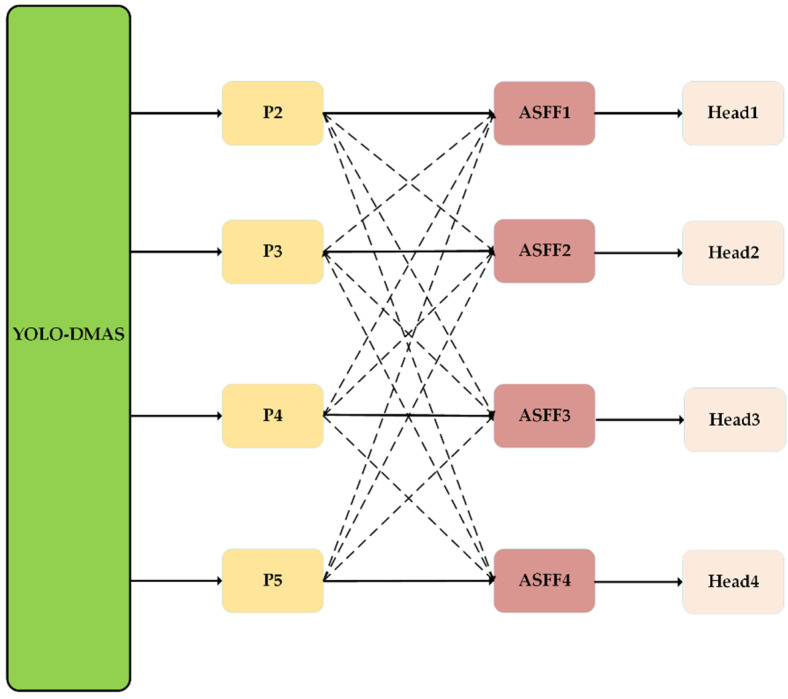
Improved detection head structure.

During training, ASFF adaptively learned the spatial fusion weights for each scale’s feature map, enabling the thorough filtration of features from other layers that carried contradictory information. This approach effectively mitigated the impact of spatial inconsistencies in feature fusion, thereby avoiding the issue of multi-scale information conflicts arising from the presence of differently sized weeds in the detection image. The architecture of the enhanced ASFF detection head, as depicted in Figure 10, benefited from the continuous adjustment of weight coefficients through back-propagation, significantly enhancing the model’s capability for multi-scale detection.

#### 3.3.4. Soft-NMS Method

Non-maximum suppression (NMS) was an important part of the target detection process. It is generally used in the validation phase after the training is completed. NMS sorted the proposal boxes from highest score to lowest score first and then selected the proposal box with the highest score. Other boxes with significant overlap with the selected box were suppressed. This process was recursively applied to other detection boxes, ensuring that each target corresponded to a detection box.

Many difficult samples with dense and partially obscured weeds were present in our dataset. When two target frames were very close to each other, the frame with a lower score, according to the NMS algorithm, would be erroneously removed because of the large overlapping area. This leads to many false detections, which would seriously affect the detection performance of the model. For this reason, we replaced the traditional NMS method with Soft-NMS [39]. The concerns arising from dense detection could be handled more accurately.

When the overlap degree of the candidate boxes is greater than the threshold, NMS will directly delete the candidate boxes. Unlike NMS, SoftNMS used an attenuation function to reduce the score of overlapping boxes and retain some boxes that would be suppressed. Mathematically expressed in Equation (8) as follows:

(8) $$s_{i}\leftarrow s_{i}f(iou(M,b_{i}))$$

where M was the current highest score, $b_{i}$ was the box to be processed, and $s_{i}$ was the score of the ith proposal box. $f(iou\left(M,b_{i}\right))$ was a weight function. Two function representations were generally used. The first was a linear decay function, which was mathematically demonstrated in Equation (9) as follows:

(9) $$s_{i}=\left\{\begin{array}{l} s_{i},\;\;IoU(M,b_{i})<N_{t}\\ s_{i}(1-IoU(M,b_{i})),IoU(M,b_{i})\geq N_{t} \end{array}\right.$$

where $\mathrm{IoU}(M,b_{i})$ was the Intersection of the Union of M and $b_{i}$.$N_{t}$ was the threshold of NMS, and the CIoU loss function was used by default.

When the IoU was larger, the score would be smaller, which leads to linear decay equivalent to ordinary NMS in the extreme case. Discontinuities in the function also caused breaks in the scores in the bbox set. Therefore, a second Gaussian decay function was used to replace the NMS, which was calculated by Equation (10):

(10) $$s_{i}=s_{i}e^{-\frac{IoU(M,b_{i}{)}^{2}}{\sigma }},\;\;\forall b_{i}\notin D$$

where σ was the parameter to adjust the Gaussian distribution center.

Utilizing a Gaussian function offered the advantage of creating a smooth gradient within a specific range. The intensity of the penalty increased as one moved closer to the center of the Gaussian distribution. By adjusting the σ parameter, the center of the distribution could be shifted, ensuring continuity through an exponential formulation. This technique effectively reduced the likelihood of false detections common with traditional NMS, enhancing the detection of dense and partially obscured weeds in complex cotton field scenarios and bolstering the model’s ability to generalize across challenging samples.

## 4. Conclusions

In this study, we developed the YOLOv8-DMAS cotton weed detection model based on an enhanced cotton weed dataset to address the challenges of identifying various weed species, different sizes of weeds, and achieving accurate dense detection. By substituting the Bottleneck structure in the C2f with the DWR module and incorporating a Multi-Scale module following the SPPF, we significantly enhanced the model’s performance in multi-scale target detection. Additionally, we integrated a small target detection head and the ASFF mechanism into the original detection framework to improve spatial consistency and the detection accuracy of small-sized targets. By replacing the conventional NMS with SoftNMS, we further mitigated issues related to false detections in dense scenarios. Experimental results demonstrated that our model surpassed other leading algorithms, achieving 95.5% and 92.1% for mAP0.5 and mAP0.5:0.95, respectively, marking improvements of 2.1% and 3.7% over YOLOv8s. Compared to models in other studies that can only identify a limited number of weed species with similar sizes and struggle with dense and partially occluded scenarios, the YOLOv8-DMAS algorithm introduced in this study represents a novel approach to detecting multiple species of cotton weeds and facilitating fast and precise detection in complex cotton field environments, even on mobile devices with limited computational capacity. This provided substantial theoretical backing for the automated eradication of cotton weeds. In the future, our method would explore its applicability to the detection of other crops, extending its use beyond cotton fields. This would involve validating our model across various agricultural environments, thereby enhancing its robustness and generalizability. Furthermore, while the model’s detection time satisfied real-time detection requirements, there remains potential for further optimization when deployed in actual cotton field environments. The next focus of this research would be on model lightweighting to enhance its deployment efficiency on edge devices.

## Figures and Tables

**Table 1:** Comparison of ablation experiment results (D: C2f_DWR; M: Multi-scale Block; A: ASFF-Tiny Detect; S: Soft-NMS).

D	M	A	S	P (%)	R (%)	mAP0.5 (%)	mAP0.5:0.95 (%)	Params (M)	GFlops (G)
×	×	×	×	94.1	88.4	93.4	88.4	11.2	28.6
√	×	×	×	94.9	87.5	93.9	88.9	10.6	27.4
√	√	×	×	94.3	89.6	94.7	89.7	13.2	29.5
√	√	√	×	95.3	91.1	94.5	91.4	19.03	51.2
√	√	√	√	95.8	92.2	95.5	92.1	19.03	51.2

× and √ represent the usage of each improved module in the experiment.

**Table 2:** Comparison of results of different models in the weed detection task.

Algorithm	Parameters (M)	P (%)	R (%)	mAP@0.5 (%)	mAP@0.5:0.95 (%)	Calculations (GFLOPs)	FPS (ms/Frame)
YOLOv8s	11.13	94.1	88.4	93.4	88.4	28.4	85.67
YOLOv3	61.583	88.4	88.2	91.5	83.6	154.7	80.65
YOLOv4 Tiny	6.057	94.1	79.00	89.6	61.7	6.9	92.18
YOLOv5s	7.023	93.9	88.2	93.7	87.3	15.8	85.19
YOLOv7	37.256	94.2	88.0	93.4	87.6	105.3	74.07
YOLOv8l	43.64	94.5	88.8	94.0	89.7	165.5	73.69
SSD	50.21	83.4	86.6	89.6	49.2	360.7	47.938
Faster R-CNN	137.09	77.6	95.1	94.74	74.3	370.2	10.262
Ours	19.03	95.8	92.2	95.5	92.1	51.2	82.47

**Table 3:** Detailed information on dataset categories, image size dimensions, segmentation ratios, sample sizes, and their data augmentation.

Dataset Category	Division Ratio	Sample Size	Augmented
Training set	70%	7906	√
Validation set	15%	847	×
Test set	15%	848	×

× and √ indicate whether the dataset has been data augmented or not.

## Data Availability

The data presented in this study are available in [Zenodo] at [https://doi.org/10.5281/zenodo.7535814].
